# When deception becomes easy: the effects of task switching and goal neglect on the truth proportion effect

**DOI:** 10.3389/fpsyg.2015.01666

**Published:** 2015-11-03

**Authors:** Bram Van Bockstaele, Christine Wilhelm, Ewout Meijer, Evelyne Debey, Bruno Verschuere

**Affiliations:** ^1^Department of Developmental Psychology, University of AmsterdamAmsterdam, Netherlands; ^2^Department of Child Development and Education, University of AmsterdamAmsterdam, Netherlands; ^3^Department of Clinical Psychology, University of AmsterdamAmsterdam, Netherlands; ^4^Institute of Medical Psychology and Systems Neuroscience, University of MuensterMuenster, Germany; ^5^Department of Clinical Psychological Science, Maastricht UniversityMaastricht, Netherlands; ^6^Department of Experimental-Clinical and Health Psychology, Ghent UniversityGhent, Belgium

**Keywords:** truth default, frequent lying, switch cost, inhibition, deception

## Abstract

Lying is typically more cognitively demanding than truth telling. Yet, recent cognitive models of lying propose that lying can be just as easy as truth telling, depending on contextual factors. In line with this idea, research has shown that the cognitive cost of deception decreases when people frequently respond deceptively, while it increases when people rarely respond deceptively (i.e., the truth proportion effect). In the present study, we investigated two possible underlying mechanisms of the truth proportion effect. In Experiment 1 (*N* = 121), we controlled for the impact of switch costs by keeping the number of switches between deceptive and truthful responses constant. We found that people who often responded deceptively made fewer errors when responding deceptively than people who only occasionally responded deceptively, replicating the truth proportion effect. Thus, while the truth proportion effect in earlier studies may be partially driven by the cost of switching between truthful and deceptive responses, we still found evidence for the truth proportion effect while controlling for switch costs. In Experiment 2 (*N* = 68), we assessed whether the truth proportion effect is influenced by goal neglect. According to this view, the truth proportion effect should be reduced if participants are cued to maintain the task goals, while it should be larger when participants are allowed to neglect the task goals. In line with this hypothesis, we found a smaller truth proportion effect when participants were cued with the task goals compared to when they were not cued. This study shows that the truth proportion effect is influenced by goal neglect, implying that frequent deceptive responding strengthens the goal of responding deceptively. Our findings imply that the accuracy of lie detection tests could be increased by using a majority of truth-items (i.e., induce the truth proportion effect), and that the truth proportion effect should be maximized by (1) increasing the number of truth-lie task switches and (2) inducing goal neglect.

## Introduction

Lying is commonly defined as “[making] an untrue statement with intent to deceive” (Lie, 2015). Deliberate and successful lying is generally considered to be more difficult than telling the truth. Cognitive models posit that lying requires more cognitive resources than truth telling (Gombos, 2006; Vrij et al., 2006, 2011; Blandón-Gitlin et al., 2014). These additional resources are needed—among other things—to suppress the truth, to monitor the behavior of the listener, and to fabricate and if necessary adapt the story. Several neuro-imaging studies have provided evidence in line with this idea, showing increased activation in prefrontal brain regions involved in cognitive control during deception (e.g., Christ et al., 2009; Farah et al., 2014; Gamer, 2014). It has been argued that this cognitive cost of lying is invariable (e.g., Johnson et al., 2005), implying that cognitive lie detection tests should be well able to differentiate between liars and truth-tellers. However, recent theories propose that lying is not always more difficult than telling the truth, suggesting that the cognitive cost of lying becomes larger or smaller depending on contextual factors, and thus that cognitive lie detection tests should take these factors into account (e.g., see Walczyk et al., 2013).

According to the Information Manipulation Theory II (McCornack et al., 2014), lying does not always require more cognitive resources than truth telling (see also Vrij, 2015). Depending on contextual factors and how readily information can be retrieved from memory, both lying and truth telling can be cognitively demanding or relatively easy. Lying will be more difficult than truth telling when for instance the truth is easily accessed in memory, or when constructing a lie involves the retrieval of information from long-term memory. When such conditions are reversed, lying may be easier than telling the truth. The theory further specifies that people who often attain their goals through lying will be more likely to continue to lie. For these people, lying is thought to be efficient, well-practiced, and therefore—in some cases—cognitively less demanding than telling the truth. A similar view on lying is also presented in the Activation-Decision-Construction-Action Theory (ADCAT; Walczyk et al., 2014). These authors specify a number of conditions under which lying is likely more difficult than truth telling, such as high motivation, or anxiety of the liar, or telling complex, unfamiliar, or unrehearsed lies. ADCAT therefore predicts that lying may be cognitively less demanding than truth telling when a lie is simple or when a lie is extensively prepared or rehearsed.

In experimental studies, the cognitive cost of lying is typically investigated using deception paradigms, in which participants are required to respond either truthfully or deceptively to relatively simple statements by pressing appropriate response buttons^1^. The results of two early studies were not in line with the idea that responding deceptively may be as demanding as responding truthfully. Johnson et al. (2005) found that practice in deceptive responding influenced neither behavioral nor neurological measures of cognitive control. In a study of Vendemia et al. (2005), the reaction time data revealed no practice effect on the cognitive cost of responding deceptively, although the error data did show that the cognitive cost of deceptive responding diminished following practice.

More recent experimental evidence is more in line with the idea that the cognitive cost of deception can be reduced through practice. Verschuere et al. (2011) used a Sheffield lie test (Spence et al., 2001) to assess the cognitive cost of deception. In this task, participants are presented brief YES–NO questions (e.g., Did you buy a newspaper today?), and they are required to respond truthfully or deceptively depending on cues (e.g., the color of the statements). The cognitive cost of deception is inferred from the difference in reaction times and error rates between lie-trials and truth-trials, and it has been repeatedly found that deceptive responses are typically slower and more error-prone than truthful responses (Verschuere et al., 2014). In order to address the effect of frequent deception on the cognitive cost of deception, Verschuere et al. manipulated the proportion of lie/truth-trials across participants. In a frequent-lie group, participants were required to respond deceptively on 75% of the trials and to respond truthfully on only 25% of the trials. Inversely, participants in a frequent-truth group responded truthfully on 75% of the trials and responded deceptively on only 25% of the trials. They found that participants in a control group with a lie/truth ratio of 50/50 were slower and made more errors on lie-trials than on truth-trials (i.e., the so-called *lie-effect*), reflecting that responding deceptively is cognitively more demanding than responding truthfully. More crucially though, this lie-effect was further enhanced in the frequent-truth group and it was reduced to near zero in the frequent-lie group. We refer to this effect as the truth proportion effect (in analogy to the proportion congruency effect; see Schmidt, 2013), because it reflects that responding deceptively becomes even more difficult when people only seldom do so, and responding deceptively becomes just as easy or difficult as responding truthfully when people frequently give deceptive responses. The results of Verschuere et al. were later replicated by Van Bockstaele et al. (2012), who showed that trained deceptive responses remained as easy or difficult as the truthful responses, even when the ratio of lie/truth-trials was set back to 50/50 after the training. Finally, Hu et al. (2012) found a near zero difference between truthful and deceptive responses in both error rates and reaction times in participants who were trained to respond deceptively, while the lie-effect was left intact in untrained participants.

Although there is evidence for the idea that the cognitive cost of deception can be influenced by manipulating the proportion of truth-trials, the underlying mechanism of the truth proportion effect is poorly understood. A thorough understanding of the mechanisms behind the truth proportion effect is imperative, as this would allow researchers to map the boundary conditions of cognition-based lie detection as well as improve the accuracy of cognitive lie detection tests. One mechanism that can account for the truth proportion effect is based on task switching (e.g., see Monsell, 2003; Vandierendonck et al., 2010). In a context where participants' specific tasks can vary on a trial-by-trial basis, performance on trial *N* depends on the task that participants performed in the preceding trial *N-1*. Performance on trial *N* is usually better (i.e., faster reaction times and less errors) when trials *N* and *N-1* require participants to perform the same task (repetition trials), while it is usually worse when trials *N* and *N-1* require the participant to perform different tasks (switch trials). The difference in performance between task repetition trials and task switch trials is referred to as switch cost. In the studies of Verschuere et al. (2011) and Van Bockstaele et al. (2012), participants were required to switch between responding deceptively and responding truthfully. For the frequent-lie group, truth-trials were relatively rare, making them more likely to be preceded by a lie-trial than a truth-trial (e.g., Lie-Lie-Truth-Lie-Lie). Truth-trials were therefore more likely to involve a task switch, whereas lie-trials more likely involved a task repetition. Conversely, in the frequent-truth group, truth-trials were more likely task repetitions and lie-trials were more likely switch trials (e.g., Truth-Truth-Truth-Lie-Truth). As such, different distributions of task switch costs over lie- and truth-trials may have increased the lie-effect in the frequent-truth group, and reduced it in the frequent-lie group.

Another possibility is that the truth proportion effect is driven by goal neglect (Duncan, 1995). According to the goal neglect theory, task goals guide the selection of appropriate responses, especially in tasks that require high levels of cognitive control. Fast and accurate responses are driven by more active task goals, while slower and more erroneous responses occur when the necessary task goals are neglected. For instance, in the Stroop task (Stroop, 1935), participants are required to respond to the ink color of color words while ignoring the meaning of the word. Typically, people are faster to respond to congruent trials, in which the ink color matches the meaning of the word (e.g., “BLUE” in blue ink), compared to incongruent trials, in which the ink color and the meaning of the word mismatch (“BLUE” in green ink). De Jong et al. (1999) manipulated goal neglect vs. goal maintenance in a Stroop task by using long vs. short response-stimulus intervals (RSIs). They argued that long RSIs would lead to lapses in attention and decreased inhibition, thus promoting goal neglect, while short RSIs would improve attentional focus and the capacity to inhibit the meaning of the words, thus promoting goal maintenance. In line with their hypothesis, they found that the Stroop effect was smaller at short RSIs compared to long RSIs. Previous research has shown that the overall cognitive cost of deception is influenced by goal neglect. Debey et al. (2012) found that lie-effects increased when participants were manipulated to neglect task goals compared to when they remained focused on the task goals. In the studies of Verschuere et al. (2011) and Van Bockstaele et al. (2012), the manipulation of the truth proportion may have induced different goals in the frequent-lie and frequent-truth groups. When participants were required to respond deceptively on most of the trials, responding deceptively was likely the most active task goal. As such, participants in the frequent-lie groups were more likely to respond fast and accurately on lie-trials rather than truth-trials, thus decreasing the size of the lie-effect. Inversely, when participants responded truthfully on most of the trials, responding truthfully was likely the most active task goal. Participants in the frequent-truth groups may therefore have been more likely to respond fast and accurately on truth-trials rather than lie-trials, resulting in an increased lie-effect.

In the present study, we experimentally addressed the role of these two possible underlying mechanisms (i.e., switch costs and/or goal neglect) of the truth proportion effect. If the truth proportion effect is driven by either (or both) of these mechanisms, this knowledge could be used to maximize the difference between deceptive and truthful responses in cognitive lie-detection tests. In both experiments, we manipulated the proportion of lie-trials and truth-trials between subjects, thus creating a frequent-lie group, a frequent-truth group, and a control group (similar to Van Bockstaele et al., 2012 and Verschuere et al., 2011). In Experiment 1, we controlled for the possible influence of switch costs by creating sequences of crucial trials in which lie-trials and truth-trials alternated consistently. These sequences of trials were alternated with sequences of only lie-trials (frequent-lie group), only truth-trials (frequent-truth group), or alternating lie- and truth-trials (control group). If the truth proportion effect is not driven by switch costs, we expected to replicate our previous findings (Verschuere et al., 2011; Van Bockstaele et al., 2012), whereas the truth proportion effect should disappear when it is driven by switch costs. In Experiment 2, we manipulated goal maintenance by adding an informative cue on half of the trials (e.g., see Parris et al., 2012). This cue indicated whether the following trial would be a lie-trial or a truth-trial. We expected that these cues would reinstate the presumed neglected goal (i.e., responding truthfully in the frequent-lie group and responding deceptively in the frequent-truth group), hence reducing the truth proportion effect.

## Experiment 1

### Methods

#### Participants

One hundred and thirty students participated in this experiment in exchange for course credits. The entire experiment took about 20 min. All participants were informed about the general nature of the tasks and signed the informed consent form before the start of the experiment. The study was approved by the ethical committee of the department of psychology of the University of Amsterdam.

#### Sheffield lie test

The Sheffield Lie Test consisted of a practice phase (60 trials) and a test phase (160 trials). The goal of the practice phase was to clarify the task instructions and get participants acquainted with the task. For this purpose, we used six questions related to general knowledge (e.g., “Is London in Germany?”, see Appendix 1 for all questions used in Experiment 1). Half of these questions required a YES response (pressing the “4” key on a normal keyboard), while the other half required a NO response (pressing the “6” key). The YES–NO response labels always remained on the screen according to the response mapping. Depending on the color of these response labels (yellow or blue), participants were required to respond truthfully or deceptively as quickly and as accurately as possible. The meaning of the colors (lie vs. truth) was counterbalanced across participants. In a first part of the practice phase, the six questions were presented twice. Participants were required to give truthful YES-responses in the first 3 trials, followed by 3 truthful NO-responses, 3 deceptive YES-responses, and 3 deceptive NO-responses. In the following 12 trials, 6 lie-trials and 6 truth-trials were presented randomly. The inter-trial interval (ITI) in these first 24 trials varied randomly between 1500, 2000, and 2500 ms. In the last 36 practice trials, 18 lie- and 18 truth-trials were presented randomly, and the ITI varied between 500, 1000, and 1500 ms. Throughout the entire practice phase, feedback was given on both correct and incorrect responses, and there was no response deadline.

In the test block, we used 40 autobiographical questions (e.g., “Do you live in the Netherlands?”). Half of these questions was repeated 4 times in filler trials, while the other half was repeated 4 times in test trials. In total, the test block consisted of 160 trials: 80 filler trials and 80 test trials. The use of the question sets in either filler or test trials was counterbalanced across participants. The filler trials were used to create 3 groups of participants that differed in their frequency of deceptive responding. In the frequent-lie group, all filler trials required a deceptive response, while in the frequent-truth group, all filler trials required a truthful response. For the filler trials in the control group, we wanted to present 40 lie-trials and 40 truth-trials. However, due to a small programming error, we presented 43 lie-trials and 37 truth-trials. In all 3 groups, the test trials consisted of 40 lie-trials and 40 truth-trials. Hence, out of a total of 160 trials, participants in the frequent-lie group were required to respond deceptively on 120 trials (75%), participants in the frequent-truth group responded deceptively on 40 trials (25%), and participants in the control group responded deceptively on 83 trials (52%). Filler and test trials were presented in alternating sequences of 10 trials (i.e., 10 filler trials, 10 test trials, 10 filler trials, 10 test trials, and so on). In order to control for switch costs, each sequence of test trials in all 3 groups consisted of 5 lie-trials and 5 truth-trials in alternating order (i.e., lie-truth-lie-truth-etc.). The ITI varied randomly between 500, 1000, and 1500 ms, and all trials had a response deadline of 3000 ms. No feedback was given in the test phase. The entire task was programmed using Inquisit 3 (2010), and it was run on a standard Windows computer.

### Results

#### Data preparation and outliers

First, we discarded the data of the practice phase. Trials with responses faster than 300 ms (0.7%) and equal to 3000 ms (response deadline: 2.0%) were removed. Next, we calculated the overall error rate in the entire block as well as the error rate for filler and test trials separately. Based on these error rates, we removed participants who made too many errors and were likely not complying with the task instructions. Participants who made more than 30% errors in total (*N* = 8) or more than 40% errors on test trials (*N* = 1) were removed from the data, resulting in a final sample of 121 participants. For the analysis of the error rates, we then removed the filler trials as well as the first trial in each test trial sequence, because these first switch trials after long repetition sequences are likely to result in abnormally high switch costs. Finally, we calculated the error rate for lie- and truth-trials separately. For the RT analyses, we first removed trials with errors (16.0%). Next, we removed all trials with RTs deviating more than 3 *SD*s from the overall average group RT (*M* = 1342 ms, *SD* = 474, removal of 0.8% of the remaining data), as well as trials with RTs deviating more than 3 *SD*s from each individual's average RT (0.8%). Finally, we removed the filler trials and the first trial of each test trial sequence, and we calculated mean RTs for lie- and truth-trials.

#### Error rates

We subjected the error rates to a 2 (Deception: lie vs. truth) × 3 (Group: frequent-lie vs. control vs. frequent-truth) repeated measures ANOVA, with Deception as a within-subjects factor and Group as a between-subjects factor. This analysis yielded a significant main effect of Deception, *F*_(1, 118)_ = 30.95, *p* = 0.000, *f* = 0.51, indicating that participants made more errors on lie-trials (*M* = 18.91%, *SE* = 0.89) than on truth trials (*M* = 14.57%, *SE* = 0.81), and a significant interaction between Deception and Group, *F*_(2, 118)_ = 4.78, *p* = 0.01, *f* = 0.28 (see Table 1)^2^. The main effect of Group was not significant, *F*_(2, 118)_ = 1.94, *p* = 0.15. To follow up on the significant interaction, we calculated lie-effect scores by subtracting the error rate for truth-trials from the error rate for lie-trials. Between-group comparisons revealed a larger lie-effect in the frequent-truth group compared to the frequent-lie group, *F*_(1, 79)_ = 8.56, *p* = 0.004, *d* = 0.63, and a marginally larger lie-effect in the frequent-truth group compared to the control group, *F*_(1, 81)_ = 3.77, *p* = 0.06, *d* = 0.41. There was no significant difference between the frequent-lie group and the control group, *F*_(1, 76)_ = 1.51, *p* = 0.22, *d* = 0.28. The difference between lie- and truth-trials was significant in the control group, *F*_(1, 39)_ = 10.88, *p* = 0.002, *f* = 0.53, and the frequent-truth group, *F*_(1, 42)_ = 34.37, *p* = 0.000, *f* = 0.90, but not in the frequent-lie group, *F*_(1, 37)_ = 1.02, *p* = 0.32, *f* = 0.17. As such, these results suggest that the truth proportion effect is still apparent when switch costs are kept constant across groups.

**Table 1:** **Errors and reaction times in Experiment 1**.

		**Frequent-lie (*N* = 38)**		**Control (*N* = 40)**		**Frequent-truth (*N* = 43)**	
		***M***	**(*SD*)**	***M***	**(*SD*)**	***M***	**(*SD*)**
Errors (%)	Lie	16.45	(8.72)	17.56	(9.44)	22.35	(10.17)
	Truth	14.91	(10.01)	13.66	(8.09)	15.12	(8.86)
	Lie effect	1.54	(9.42)	3.90	(7.48)	7.23	(8.09)
RTs (ms)	Lie	1499	(249)	1520	(258)	1389	(213)
	Truth	1411	(246)	1426	(224)	1332	(199)
	Lie effect	88	(136)	95	(125)	57	(132)

#### Reaction times

A similar 2 (Deception) × 3 (Group) repeated measures ANOVA on the RT data revealed a significant main effect of Deception, *F*_(1, 118)_ = 44.86, *p* = 0.000, *f* = 0.62, indicating that participants responded slower on lie-trials (*M* = 1467 ms, *SE* = 22) than on truth-trials (*M* = 1387 ms, *SE* = 20). The main effect of Group was also significant, *F*_(2, 118)_ = 3.10, *p* = 0.05. Between-group comparisons showed that participants in the frequent-truth group (*M* = 1360 ms, *SE* = 30) were faster than participants in the control group (*M* = 1473 ms, *SE* = 37), *F*_(1, 81)_ = 5.72, *p* = 0.02, and marginally faster than participants in the frequent-lie group (*M* = 1455 ms, *SE* = 39), *F*_(1, 79)_ = 3.88, *p* = 0.05, with no difference between the control group and the frequent-lie group, *F* < 1. The crucial interaction was not significant, *F* < 1, indicating that the truth proportion effect was not present in the RT data when the effects of task switching were controlled for (see Table 1). In other words, the standard lie-effect was present in all 3 groups [frequent-lie group: *F*_(1, 37)_ = 15.97, *p* = 0.000, *f* = 0.66; control group: *F*_(1, 39)_ = 22.76, *p* = 0.000, *f* = 0.76; frequent-truth group: *F*_(1, 42)_ = 8.03, *p* = 0.007, *f* = 0.44], but there was no difference between the 3 groups. In sum, the RT data suggest that the truth proportion effect is to a certain degree dependent upon switch costs.

### Discussion

In Experiment 1, we kept the number of switches on lie- and truth-trials equal to investigate whether the previously reported truth proportion effect was driven by differences in switch costs. If this were the case, we expected that the truth proportion effect would disappear, whereas we expected to replicate the truth proportion effect if it is not driven by switch costs. Our results show that there was no sign of a truth proportion effect in the RT data, with all groups showing similar increased RTs on lie-trials relative to truth-trials. In contrast, the error data did still reveal a truth proportion effect, as illustrated by the larger lie-effect in the frequent-truth group relative to the frequent-lie group, and a similar tendency when comparing the frequent-truth group, and the control group. Hence, the RT data indicate that the truth proportion effect is to a certain degree driven by switch costs, but the largely intact truth proportion effect in the error data suggests that differences in switch costs are likely not the only phenomenon that contributes to the truth proportion effect.

## Experiment 2

### Methods

#### Participants

Seventy-two students participated in this experiment in exchange for either course credits or €3.5. The entire experiment took about 20 min. All participants were informed about the general nature of the tasks and signed the informed consent form before the start of the experiment. The study was approved by the ethical committee of the department of psychology of the University of Amsterdam

#### Sheffield lie test

The overall appearance of the Sheffield Lie Test was identical to the test used in Experiment 1. The test consisted of two practice blocks and one test block. In the practice blocks, we used 12 questions about semantic knowledge (e.g., “Is there water in the sea?”, see Appendix 2 for all questions used in Experiment 2), 6 of which required YES-responses and 6 required NO-responses. The first practice block consisted of 3 truthful YES-responses, followed by 3 deceptive NO-responses, 3 truthful NO-responses, and 3 deceptive YES-responses. The second test block consisted of 24 trials (6 deceptive YES, 6 deceptive NO, 6 truthful YES, and 6 truthful NO), presented in random order. In both practice blocks, feedback was given on correct and incorrect responses, and the ITI was 0 ms.

In the test block, we used a new set of 36 semantic YES-NO questions. Each question was presented 8 times: 4 times in filler trials and 4 times in test trials, for a total of 288 trials. The filler trials consisted of all lie-trials (frequent-lie group), all truth-trials (frequent-truth group), or 50% lie- and 50% truth-trials (control group). The test trials consisted for all 3 groups of 50% lie- and 50% truth-trials. As such, 75% of the trials in the frequent-lie group were lie-trials, compared to 50% in the control group and 25% in the frequent-truth group. In order to manipulate goal maintenance, half of the filler and test trials were preceded by an informative cue that was presented for 1 s in the center of the screen prior to the presentation of the question. The cue was either “LIE” or “TRUTH,” and always correctly indicated which action participants would have to perform on the following trial. The other half of all trials (filler and test trials) were not cued, and merely showed a fixation cross for 1 s. The ITI was 0 ms. There was no response deadline and no feedback was given in the test phase. The entire task was programmed using Inquisit 3 (2010), and it was run on a standard Windows computer.

### Results

#### Data preparation and outliers

First, we discarded the data of the practice phase. Trials with responses faster than 300 ms (guesses: 0.8%) were removed. Next, we calculated the overall error rate in the entire block as well as the error rate for filler and test trials separately. Participants who made more than 30% errors in total (*N* = 4) or more than 40% errors on test trials (*N* = 0) were removed from the data. For the analysis of the error rates, we then selected the test trials and calculated the error rate for lie- and truth-trials, for cued and non-cued trials separately. For the RT analyses, we first removed trials with errors (10.9%). Next, we removed all trials with RTs deviating more than 3 *SD*s from the mean group RT (*M* = 1615 ms, *SD* = 1256, removal of 1.2% of the remaining data), as well as trials with RTs deviating more than 3 *SD*s from each individual's average RT (1.9%). Finally, we removed the filler trials, and we calculated mean RTs for lie- and truth-trials for cued and non-cued trials.

#### Errors

We conducted a 2 (Deception: lie vs. truth) × 2 (Cue: cued vs. non-cued) × 3 (Group: frequent-lie vs. control vs. frequent-truth) repeated measures ANOVA on the error percentages, with Deception and Cue as within-subjects factors and Group as a between-subjects factor. This analysis revealed main effects of Deception, *F*_(1, 65)_ = 31.87, *p* = 0.000, *f* = 0.70, and Cue, *F*_(1, 65)_ = 10.71, *p* = 0.002, *f* = 0.41. Overall, participants made more errors on lie-trials (*M* = 10.95, *SE* = 0.73) than on truth-trials (*M* = 7.20, *SE* = 0.56, and they made less errors on cued trials (*M* = 8.10, *SE* = 0.58) than on non-cued trials (*M* = 10.05, *SE* = 0.65). The interaction between Deception and Group was also significant, *F*_(2, 65)_ = 9.18, *p* = 0.000, *f* = 0.53, reflecting the original truth proportion effect. To follow up on the Deception by Group interaction, we calculated lie-effect scores by subtracting the error percentage on truth-trials from the error percentage on lie-trials (irrespective of Cue). Between-group comparisons showed that the overall lie-effect was larger in the frequent-truth group (*M* = 7.28, *SD* = 6.93) than in the frequent-lie group (*M* = 0.73, *SD* = 3.70) and the control group (*M* = 2.83, *SD* = 4.45), *F*_(1, 43)_ = 15.03, *p* = 0.000, *d* = 1.18, and *F*_(1, 45)_ = 6.81, *p* = 0.01, *d* = 0.76, respectively. The frequent-lie group did not differ significantly from the control group, *F*_(1, 42)_ = 2.88, *p* = 0.10, *d* = 0.52. This overall lie-effect (irrespective of Cue) was significant in the control group, *F*_(1, 22)_ = 9.47, *p* = 0.006, and the frequent-truth group, *F*_(1, 23)_ = 26.61, *p* = 0.000, but not in the frequent-lie group, *F* < 1, *p* = 0.38. The crucial Three-way interaction between Cue, Deception, and Group was not significant (see Table 2), *F*_(2, 65)_ = 1.97, *p* = 0.15, *f* = 0.25, nor were any other effects, all *F*s < 1. Exploratory between-group comparisons of the lie-effect scores for cued and non-cued trials separately revealed no group differences on cued trials, all *F*s < 2.10, all *p*s >0.15. For non-cued trials, the lie-effect was larger in the frequent-truth group compared to both the control group and the frequent-lie group, both *F*s > 6.38, both *p*s < 0.05, with no difference between the control group and the frequent-lie group, *F*_(1, 42)_ = 2.61, *p* = 0.11.

**Table 2:** **Errors and reaction times on cued and non-cued trials in Experiment 2**.

		**Frequent-lie (*N* = 21)**		**Control (*N* = 23)**		**Frequent-truth (*N* = 24)**	
		***M***	**(*SD*)**	***M***	**(*SD*)**	***M***	**(*SD*)**
Errors (%) cued	Lie	9.28	(6.30)	8.28	(5.25)	11.45	(7.74)
	Truth	7.48	(6.75)	5.64	(5.56)	6.44	(4.55)
	Lie effect	1.80	(6.18)	2.64	(7.78)	5.00	(8.35)
Errors (%) non-cued	Lie	9.63	(5.43)	10.85	(6.31)	15.70	(9.82)
	Truth	9.99	(5.81)	7.82	(6.64)	6.18	(4.66)
	Lie effect	−0.36	(5.79)	3.03	(7.87)	9.52	(9.60)
RTs (ms) cued	Lie	1598	(379)	1587	(280)	1399	(302)
	Truth	1432	(354)	1426	(308)	1272	(287)
	Lie effect	166	(187)	161	(156)	126	(219)
RTs (ms) non-cued	Lie	1607	(385)	1705	(281)	1578	(351)
	Truth	1590	(337)	1508	(286)	1267	(271)
	Lie effect	16	(201)	197	(154)	311	(208)

#### Reaction times

As for the error rates, we used a 2 (Deception) × 2 (Cue) × 3 (Group) repeated measures ANOVA on the reaction time data. This analysis revealed significant main effects of Deception, *F*_(1, 65)_ = 76.70, *p* = 0.000, *f* = 1.09, with faster RTs on truth-trials (*M* = 1410, *SE* = 38) compared to lie-trials (*M* = 1577, *SE* = 39), and Cue, *F*_(1, 65)_ = 55.56, *p* = 0.000, *f* = 0.92, with faster RTs on cued trials (*M* = 1449, *SE* = 38) than on non-cued trials (*M* = 1539, *SE* = 38), as well as a significant interaction between Deception and Group, *F*_(2, 65)_ = 4.00, *p* = 0.02, *f* = 0.35. The main effect of Group was only marginally significant, *F*_(2, 65)_ = 2.72, *p* = 0.07, and the interactions between Cue and Group and between Deception and Cue were not significant, both *F*s < 1. Crucially, the Three-way interaction between Group, Deception, and Cue was significant, *F*_(2, 65)_ = 12.70, *p* = 0.000, *f* = 0.63 (see Table 2). To follow up on this interaction, we calculated lie-effect scores by subtracting RTs on truth-trials from RTs on lie-trials for cued and non-cued trials separately. There were no differences in lie-effects between the groups on cued trials, *F* < 1, and the lie-effect was present in all 3 groups (frequent-lie group: *F*_(1, 20)_ = 16.58, *p* = 0.001, *f* = 0.91; control group: *F*_(1, 22)_ = 24.51, *p* = 0.000, *f* = 1.06; frequent-truth group: *F*_(1, 23)_ = 7.98, *p* = 0.01, *f* = 0.59). On non-cued trials, the group difference was significant, *F*_(2, 65)_ = 13.74, *p* = 0.000. Between-group comparisons revealed larger lie-effects in the frequent-truth group compared to both the control group, *F*_(1, 45)_ = 4.55, *p* = 0.04, *d* = 0.62, and the frequent-lie group, *F*_(1, 43)_ = 23.23, *p* = 0.000, *d* = 1.44, and a larger lie-effect in the control group than in the frequent-lie group, *F*_(1, 42)_ = 11.31, *p* = 0.002, *d* = 1.01. The lie-effect on non-cued trials was present in the control group, *F*_(1, 22)_ = 37.71, *p* = 0.000, *f* = 1.31, and the frequent-truth group *F*_(1, 23)_ = 53.79, *p* = 0.000, *f* = 1.53, but not in the frequent-lie group, *F* < 1, *p* = 0.71, *f* = 0.08. These results clearly indicate that the truth proportion effect disappeared on cued trials but is replicated on non-cued trials. In other words, the truth-proportion effect disappeared when participants were encouraged to maintain their task goals, illustrating that the original truth proportion effect is at least in part caused by goal neglect.

### Discussion

In Experiment 2, we manipulated goal maintenance and goal neglect by presenting cues that either had information value with regard to the upcoming task (responding truthfully vs. responding deceptively) or had no information value (a fixation cross). The RT data revealed that the truth proportion effect vanishes when participants are cued with the relevant task goal. When no such cue was presented we replicated the original truth proportion effect, finding a large lie-effect in the frequent-truth group, a medium lie-effect in the control group, and a near zero lie-effect in the frequent-lie group. This finding was only partially reflected in the error data, where the crucial Three-way interaction failed to reach significance. However, even in the error data there was a tendency for the truth proportion effect to be reduced when the correct task goals were cued. As such, the results of Experiment 2 indicate that goal neglect is one of the underlying mechanisms of the truth proportion effect.

## General discussion

Although lying is generally considered to be more cognitively demanding than truth telling, recent models proposed that the cognitive cost of lying is influenced by contextual factors. In order to fully develop a cognitive approach to lying and lie-detection, these contextual factors need to be uncovered. Recent studies have shown that the cognitive cost of deception depends on the proportion of lie/truth-trials (Verschuere et al., 2011; Van Bockstaele et al., 2012). In the present study, we examined whether this truth proportion effect is driven by task switching (Experiment 1) and/or goal neglect (Experiment 2). When controlling for switch costs, the truth proportion effect disappeared in the RT data but was still maintained in the error data. Manipulating goal neglect had strong effects on the truth proportion effect: When goal neglect was reduced, the truth proportion effect also diminished, while the truth proportion effect was left intact when goal neglect was not reduced. This was especially the case in the RT data and only marginally so in the error data. Taken together, our findings suggest that the original truth proportion effect may be partially driven by switch costs and is most likely influenced by goal neglect.

The replication of the original truth proportion effect in the error data of Experiment 1 and in the non-cued trials of Experiment 2 further adds to the idea that lying is not always more cognitively demanding than truth telling (McCornack et al., 2014; Walczyk et al., 2014; Vrij, 2015), as in these conditions there was no significant lie-effect in the frequent-lie group. As such, lying may be just as easy or difficult as truth telling when people lie frequently or when lies are well-practiced, although it should also be noted that we found no evidence for the idea that deceptive responding can be *easier* than truthful responding. This finding has important implications for the detection of deception. On the one hand, it suggests that people can train themselves to become practiced liars who will experience little if any difficulty when responding deceptively, which would most often lead to inconclusive (but not false negative) results on RT-based lie-detection tests. On the other hand, it implies that lie detection may be improved by asking suspects a large majority of verifiable questions on which they have to respond truthfully, hence increasing the cognitive cost of deception on a minority of critical incriminating questions.

A limitation of this approach concerns the generalizability of these findings to new items and new settings. Van Bockstaele et al. (2012) showed that responding deceptively in general was more difficult *while* participants were more often responding truthfully (i.e., in the training phase), yet it only *remained* more difficult for those specific items on which participants always responded truthfully in the training phase. As we used the same items for filler and test trials in Experiment 2, it is possible that these findings were item-specific and will not necessarily generalize to new items. Nevertheless, our results indicate that introducing a relatively higher number of truth-trials adds to the list of cognitive load manipulations that may improve the detection of liars. Vrij et al. (2011), for instance, proposed that creating task demands that increase cognitive load (e.g., by asking suspects to tell their story in reversed order) will make the interview more difficult, especially so for lying suspects. In a similar vein, interviewers could ask unexpected questions, for which a suspect is unlikely to have prepared and practiced a lie (e.g., see Vrij et al., 2009). In contrast to many deception studies that proposed cognitive load manipulations without exploring their underlying mechanisms, the current study highlights switch costs and goal neglect as potential drivers of the truth proportion effect. Such a process-oriented investigation validates the use of this cognitive manipulation for lie detection purposes and enhances further theoretical understanding of deception.

An interesting issue concerns how our findings of proportion effects in deception tasks relate to the literature on cognitive control and the resolution of conflict. In the Sheffield lie test, there is a conflict between truthful and deceptive responding, leading to the general lie-effect. In a similar vein, people experience conflict in the Stroop task, where they are required to respond to the ink-color of color-words, the flanker task (Eriksen and Eriksen, 1974), where a central target is flanked by task-relevant distracters (e.g., > > < > >), or the Simon task (Simon and Rudell, 1967), where participants respond with right or left button presses based on for instance the color of targets that are presented on the right or left side of the screen. These conflict tasks have all been shown to be influenced by congruency proportion effects (Bugg and Crump, 2012). For instance, the Stroop effect (i.e., the RT for incongruent minus congruent trials) is reduced when the majority of the trials are incongruent (e.g., the word BLUE printed in red), and it is increased when the majority of the trials is congruent (i.e., the word RED printed in red). Traditionally, such proportion effects have been explained by the conflict adaptation hypothesis (e.g., Botvinick et al., 2001). This hypothesis assumes that participants detect and use the congruency proportion, leading them to pay more (vs. less) attention to the meaning of the words rather than their color in the condition with mostly congruent (vs. mostly incongruent) trials. Schmidt (2013) formulated an alternative, bottom-up idea. According to his *contingency* hypothesis, people use the identity of the stimulus to predict which response is most likely to be appropriate. If trials are mostly congruent, the identity of the word BLUE predicts that the correct response is most likely “blue.” If most trials are incongruent, the identity of the word BLUE predicts that the correct response is unlikely “blue,” but rather another color. In the context of our deception experiments (Verschuere et al., 2011; Van Bockstaele et al., 2012, Experiment 1 of the current study), the contingency account cannot fully explain the truth proportion effect. In these experiments, the truth proportion was manipulated by an independent set of filler trials, and the lie-effect was calculated for the test trials only. For the test trials, the contingency between the stimuli and the responses did not vary with truth proportion condition. Hence, participants could not use individual stimuli to predict whether the most likely task would be to respond deceptively or responding truthfully. An explanation in terms of conflict adaptation may therefore be more likely to explain the truth proportion effect. In the high truth proportion condition, participants may have noticed that their automatic response (i.e., the truth) mostly resulted in the correct response, and hence decided to “follow their gut.” In the low truth proportion condition, the automatic truth response mostly conflicted with the required deceptive response, and hence participants may have changed gears and performed the task in a slower, more strategic fashion.

Another issue concerns possible alternative explanations for the truth proportion effect, besides differences in task switching and goal neglect. One such alternative that we did not investigate in this study is the oddball-effect (e.g., see Squires et al., 1975; Stevens et al., 2000; Goldstein et al., 2002). In oddball tasks, participants respond to one highly frequent and one less frequent type of stimulus. Responses for the frequent stimuli are typically very fast, while responses for the infrequent stimuli are usually slower. In experiments using truth proportion manipulations, lie-trials are more frequent than truth-trials for participants in the frequent-lie group. As such, these participants should be more likely to respond fast on lie-trials and slow on truth-trials, resulting in a decreased lie-effect. Inversely, in a frequent-truth group, there are more truth-trials than lie-trials, resulting in faster responses on truth-trials compared to lie-trials, and thus a larger lie-effect. In order to investigate whether the oddball-effect also adds to the truth proportion effect, one could compare the lie-effects of crucial trials in sequences of filler trials. For instance, one could present the sequence Lf-Tf-Tf-Tf-Tc-Lc and the sequence Tf-Tf-Lf-Tf-Tc-Lc, in which L stands for lie-trial, T stands for truth-trial, c stands for crucial trial, and f stands for filler-trial. In both cases the proportion of lie- and truth-trials is the same. However, Lc in the first sequence is more of an oddball as Lc in the second sequence, as it is preceded by four consecutive truth-trials, compared to only two consecutive truth-trials in the second sequence. Hence, if the oddball effect adds to the truth proportion effect, the lie-effect (Lc minus Tc) should be larger in the first sequence than in the second sequence.

Our study is not without limitations. For instance, we did not include manipulation checks for our goal neglect induction. Another limitation concerns the ecological validity of our study. In our deception paradigm, participants were instructed to respond deceptively or truthfully on relatively simple yes-no questions by pressing one of two response buttons. This controlled set-up allowed us to manipulate and address the influence of task switching and goal neglect on the truth proportion effect. However, in more realistic settings, lying often involves the choice regarding whether or not, and how, to generate a message that manipulates truthful information, as well as social interactions, more complex stories, large gains or losses, and more emotional involvement and arousal. These more realistic circumstances differ from the binary yes/no responding in our study. Consequently, it remains to be seen whether the implications of our study also generalize to more complex applied settings, such as interviews or lie detection tests, in which people are typically faced with open-ended questions that require more elaborate responses and more cognitive effort.

Another limitation is inherent to our goal neglect manipulation. Whereas Parris et al. (2012) used general cues (e.g., “fast” or “quick”) to prime task goals, our cues were more specific in the sense that they predicted whether participants would be responding deceptively vs. responding truthfully on the following trial. Because the cues were informative of the task, our goal maintenance manipulation may have also had an effect on task switch costs. Switch costs are typically smaller when participants have more time to prepare the next task (i.e., when the Cue-Stimulus Interval is long; Meiran, 1996). On cued trials, the task cue was presented for 1 s before the stimulus sentence was presented, thus allowing participants to prepare the upcoming task. On non-cued trials, there was no such task cue, and participants only knew which task to perform upon seeing the (color of the) stimulus sentence. Furthermore, the amount of information that is presented in cues also influences switch costs (Miyake et al., 2004). Explicit cues such as ours (i.e., “Lie” and “Truth”) typically result in smaller switch costs compared to more arbitrary cues (e.g., “X” as a cue for responding deceptively and Y as a cue for responding truthfully). Our goal maintenance manipulation may thus have been confounded with differences in switch costs. However, the results of Experiment 1—in which we explicitly controlled for switch costs—showed that task switching likely has only a limited impact on the truth proportion effect, as the truth proportion effect remained intact in the error rates. Nevertheless, it seems worthwhile to replicate the present study using cues that are not informative of the task to manipulate goal neglect.

In sum, the replication of the standard lie-effect advocates the use of cognitive lie detection tests. The replication of the truth proportion effect confirms that the cognitive cost of deception is influenced by the frequency of deception. This implies that well-trained lies may be hard to differentiate from the truth, but also that lie detection tests can be improved by adding a large number of truth-items. Finally, the present experiments indicate that the truth proportion effect is likely in large part driven by goal neglect, while task switching only has a smaller influence on the truth proportion effect.

### Conflict of interest statement

The authors declare that the research was conducted in the absence of any commercial or financial relationships that could be construed as a potential conflict of interest.

## Footnotes

^1^It is important to note here that these deception paradigms do not involve the actual production of deceptive messages in a communication context. Although they do cover important aspects of lying (i.e., giving responses participants know to be false), they do not capture the full complexity of lying and thus represent only simplified and limited laboratory approximations of lying.

^2^Effect sizes for between-group contrasts were estimated with Cohen's *d*. According to Cohen (1992), values from 0.20 represent small effects, values from 0.50 represent medium effects and values of 0.80 and larger represent large effects. Effect sizes for within-subjects effects and interactions were estimated using Cohen's *f*, with values from 0.10 representing small effects, values from 0.25 representing medium effects and values from 0.40 representing large effects. We calculated *f* using the following formula: $f=\surd [\eta_{p}^{2}∕(1-\eta_{p}^{2}$)].

## Supplementary material

### Data availability statement

The original data that were used in Experiment 1 and Experiment 2 are accessible via the following link to the Open Science Framework data deposit: https://osf.io/qgsv3/?view_only=32cb9693d1b94507a5ba2952a8fe9435.
