# Supplementary material for: When deception becomes easy: the effects of task switching and goal neglect on the truth proportion effect
Source: Front Psychol. 2015 Nov 3;6:1666. doi: 10.3389/fpsyg.2015.01666 (PMC4630537; doi:10.3389/fpsyg.2015.01666)
Supplement: Supplementary file 1 [file DataSheet1.DOCX]

Appendix 1. Questions used in Experiment 1

| Practice items | Test/Filler items set 1 | Filler/Test items set 2 |
| --- | --- | --- |
| Is Brussels in Belgium? | Are you sitting in front of a computer? | Are you two years old? |
| Is water wet? | Are you European? | Are you indoors? |
| Are giants tall? | Are you asleep? | Do you live in the Netherlands? |
| Is London in Germany? | Is the screen black? | Do you have eyes? |
| Are giants small? | Are you awake? | Are you using a computer? |
| Is fire wet? | Is it the year 2009? | Are you a professor? |
|  | Are you in Ireland? | Are you Norwegian? |
|  | Is it spring? | Do you know what a Mimikus is? |
|  | Can you fly? | Do you speak a little Kazak? |
|  | Have you finished this experiment? | Do you study medicine? |
|  | Are you a student? | Is it February? |
|  | Are you watching a screen? | Are you using a keyboard? |
|  | Do you know what a dog is? | Do you have a nose? |
|  | Do you speak a little English? | Do you see words on the screen? |
|  | Are you studying in Amsterdam? | Is it winter? |
|  | Are you sitting in front of an oven? | Are you 40 years old? |
|  | Are you watching the television? | Are you sitting outside? |
|  | Do you have a doctoral degree? | Do you live in Spain? |
|  | Is it 30 degrees Celsius outside? | Do you see flowers on the screen? |
|  | Do you have a secondary school degree? | Are you using a hoover at this moment? |

Appendix 2. Questions used in Experiment 2

| Practice items | Test/Filler items |
| --- | --- |
| Do fish have scales? | Is Brussels in Belgium? |
| Do cows give milk? | Do zebras have stripes? |
| Can birds fly? | Do cars have four wheels? |
| Is there water in the sea? | Are giants tall? |
| Do books have pages? | Is fire hot? |
| Do dogs have four legs? | Is water wet? |
| Is Berlin in France? | Are rocks hard? |
| Are dolphins furry? | Are bananas yellow? |
| Do fish have two legs? | Is grass green? |
| Do fish swim in the grass? | Do sausages contain meat? |
| Are bears green? | Do chickens lay eggs? |
| Are gnomes tall? | Does a table have legs? |
|  | Is the earth round? |
|  | Can you look through a window? |
|  | Is milk white? |
|  | Is water liquid? |
|  | Is Amsterdam in the Netherlands? |
|  | Can you make phone calls with a mobile? |
|  | Is London in Germany? |
|  | Are there five seasons in a year? |
|  | Do bicycles have three wheels? |
|  | Are apples white? |
|  | Is lettuce meat? |
|  | Are elephants blue? |
|  | Are giants small? |
|  | Is it cold on the equator? |
|  | Is ice warm? |
|  | Is fire wet? |
|  | Can pigs fly? |
|  | Are diamonds soft? |
|  | Is a wheel square? |
|  | Is bread liquid? |
|  | Are there nine days in a week? |
|  | Do cars have six wheels? |
|  | Do the Netherlands border to Hungary? |
|  | Can you make phone calls with an oven? |
